# Genetic analysis of bone morphometry and ivory vertebrae in threespine stickleback

**DOI:** 10.64898/2026.04.13.718284

**Published:** 2026-04-14

**Authors:** Veronica C. Behrens, David Lee, Julia I. Wucherpfennig, David M. Kingsley

**Affiliations:** 1 Department of Developmental Biology, Stanford University School of Medicine, Stanford, CA 94305, USA; 2 Howard Hughes Medical Institute, Stanford University School of Medicine, Stanford, CA 94305, USA

**Keywords:** QTL mapping, bone morphometry, bone microstructure, ivory vertebrae, Paget’s disease, supergenes, threespine stickleback, evolution, *Eda*, *Itm2a*, *Tnfrsf1b*, *TNFRSF11A*

## Abstract

Previous genetic studies of skeletal variation in threespine stickleback fish (*Gasterosteus aculeatus*) have focused primarily on striking morphological differences. Here, we examine the largely unexplored genetic architecture of internal bone microstructural variation between marine and freshwater stickleback. μCT X-ray analysis revealed differences in the porosity, bone thickness, and bone volume fraction within armor plates and vertebrae from a marine and freshwater stickleback. Quantitative trait locus mapping in F2 progeny from a marine × freshwater stickleback cross identified a significant locus on chromosome 4 influencing multiple aspects of armor plate internal microstructure. This locus overlaps the well-characterized *Eda* region previously known to control armor plate number and size. Co-mapping of bone microstructure could be due to pleiotropic effects of *Eda* on multiple aspects of plate development or to changes in closely linked genes including *Itm2a*, which also plays a role in bone formation. Most bone microstructure traits in vertebrae showed weak or no genetic signal, consistent with a polygenic architecture. However, we identified a highly significant locus on chromosome 17 that is strongly associated with abnormally thickened “ivory vertebrae” that occurred in 8.4% of F2 offspring. This phenotype resembles Paget’s disease in humans, and the major locus region contains *Tnfrsf1b*, the stickleback ortholog of a human Paget’s disease susceptibility gene *TNFRSF11A*. Together, our findings identify genetic loci underlying natural variation in bone microstructure in wild fish and reveal a candidate gene associated with a disease-like skeletal phenotype, highlighting stickleback as a model for studying both evolutionary and pathological bone biology.

## Introduction

How and why certain traits evolve in natural populations is a longstanding question in evolutionary biology (Owen 1848; Darwin 1859; Endler 1986; Carroll 2005; Martin and Orgogozo 2013). The threespine stickleback, *Gasterosteus aculeatus*, is a uniquely powerful vertebrate model for uncovering the genetic mechanisms underlying the evolution of skeletal traits. At the end of the last glacial period 10,000 to 20,000 years ago, stickleback from marine environments began colonizing newly-formed freshwater habitats. Confronted with similar ecological challenges, many different freshwater stickleback populations independently evolved similar traits (Bell and Foster 1994).

Despite dramatic skeletal differences, marine and freshwater stickleback retain the ability to interbreed. Genetic crosses can therefore be generated between ecotypes in the laboratory, producing first generation (F1) hybrids and second generation (F2) progeny that exhibit a range of phenotypes whose distribution can be compared to the inheritance of chromosome regions from the parents of the cross. Through quantitative trait locus (QTL) mapping using stickleback crosses, specific genetic loci have been identified that control striking morphological skeletal differences, including the complete loss or changes in size or number of dorsal spines (Howes et al. 2017; Wucherpfennig et al. 2022), lateral bony armor plates (Colosimo et al. 2004), and the pelvic girdle (Shapiro et al. 2004) in freshwater fish (Kingsley and Peichel 2006; Peichel and Marques 2017).

However, less attention has been given to bone internal microstructural differences (e.g. porosity of the bone, average size of the pores, average bone thickness) between marine and freshwater stickleback, in part because assessing these traits requires high resolution imaging techniques such as micro-computed tomography (μCT) or scanning electron microscopy (SEM). The few studies that have examined the microstructure of stickleback bones show that armor plates have a textured surface and internal structure containing heterogeneous pores (Song et al. 2010; Lees et al. 2012). Others have focused on population differences, demonstrating that the armor plates of freshwater stickleback are both more porous (Wiig et al. 2016) and have lower bone mineral density (Jamniczky et al. 2018) compared to marine populations. Armor plates are also major sites of phosphate accumulation in stickleback, and previous work has shown that genetic variation in armor plate development influences whole-body elemental ratios in wild populations (Durston and El-Sabaawi 2017). However, the genetic basis of stickleback bone internal microstructural differences remains unexplored.

Here, we conduct QTL mapping using a cross between a marine and freshwater stickleback to investigate the genetic basis of internal bone morphometric variation assessed by μCT in both armor plates and vertebrae. By including armor plates, we build on the substantial body of literature focused on the evolutionary significance of lateral armor. Indeed, most previous bone morphometric studies in stickleback have focused on armor plates, and various hypotheses have been proposed for reduced bone number and size in freshwater stickleback that could also apply to bone internal microstructure variation. For example, reduced armor could provide an advantage in the face of grappling predation by macroinvertebrates (Reimchen 1992), lower availability of ions for bone formation in some environments (Giles 1983), or reduced buoyancy requirements in freshwater (Myhre and Klepaker 2009). We also include vertebrae in our study of internal bone microstructure to examine a skeletal region that is particularly relevant to changes in bone density and the clinical problems of prevalent osteoporosis and other bone disorders in humans (Kado et al. 2003; Rachner et al. 2011; Compston et al. 2019; Nguyen et al. 2024).

## Materials and methods

### Phenotyping using μCT scans

CT scans were generated using a MicroCT42 (Scanco Medical AG) with the following parameters: 10 μm^3^ voxel size, 70 kVp, 114 μA, 8 W, 300 ms integration time, and frame averaging of 2. Whole stickleback were scanned in acrylic cylinders (20 mm diameter × 75 mm high) provided by the manufacturer filled with 70% ethanol and stabilized with tissue sponges (Epredia^™^ 8453).

For measuring bone morphometry traits, the raw scan was converted to a stack of DICOM images using the μCT Evaluation Program preinstalled on the workstation provided by the manufacturer and then transferred to an external workstation. The substack of DICOM images containing the bone-of-interest was loaded into Dragonfly version 2020.1 for Windows (Comet Technologies Canada Inc., Montreal, Canada; software available at https://dragonfly.comet.tech/) and further cropped using the clipping box. Then, Dragonfly’s Image Processing Panel was used to apply the slope map filter (scale = 10), mean shift algorithm, and isodata thresholding to segment bone from background. Voxels not belonging to the bone-of-interest were manually removed using a combination of Dragonfly’s 3D lasso tool, 3D painter tool, and 2D painter tool. Finally, Dragonfly’s Bone Analysis Extension was used to compute bone morphometry measurements in three steps.

**Step 1**: Holes in the original unfilled bone were filled (hole size of 0.3 mm or 0.1 mm for the armor plate and 2nd vertebra, respectively) followed by manual filling of larger holes missed by the algorithm.

**Step 2**: This step normally separates cortical bone from trabecular bone, but it was skipped completely in this analysis because it is unreliable for fish bones.

**Step 3**: The following measurements were computed and recorded for the bone-of-interest using 0.1 mm as the estimated mean bone thickness:

- Surface area: Surface area of the unfilled bone
- Total volume (TV): Volume of the filled bone
- Bone volume (BV): Volume of the unfilled bone
- Bone volume fraction (BV/TV): Ratio of the volume of the unfilled bone to the filled bone
- Mean bone thickness: Mean thickness of the unfilled bone, assessed in 3D. Note this is described as “Trabecular thickness” by Dragonfly’s Bone Analysis Extension.
- Mean bone separation: Mean distance between the honeycomb-like structures in the unfilled bone, assessed in 3D. This was assessed only for bones with obvious honeycomb-like internal structures including the armor plate, operculum, and cleithrum. Note this is described as “Trabecular separation” by Dragonfly’s Bone Analysis Extension.

All bone morphometry measurements except bone volume fraction (BV/TV) were standardized by multiple regression against standard length (measured by standard X-ray using Fiji version 2.0.088 (Schindelin et al. 2012)) and sex.

For scoring right pleural rib number, right epipleural rib number, frontal bone ridges, and frontal bone sculpture, the raw scan was segmented using the μCT Evaluation Program preinstalled on the workstation provided by the manufacturer to keep only voxels with bone mineral density (BMD) of at least 150 mgHA/mm^3^ (Gauss sigma = 0.8 and Gauss Support = 1). Using Image Processing Language (IPL), the resulting _SEG.AIM file was downsampled by 2X, re-thresholded, and converted to a small mesh model using the scale, threshold, and stl commands, respectively. The resulting STL file was transferred to an external workstation and viewed in 3D Slicer version 4.10.2 (Fedorov et al. 2012) as a 3D model. Frontal bone ridges and frontal bone sculpture were scored on a scale from 1–5, with 5 indicating the greatest prominence.

In cases where BMD is reported, raw attenuation values were converted to hydroxyapatite (HA) density using the following linear conversion determined by scanning a density phantom with rods of known HA density (0, 100, 200, 400, and 800 mgHA/mm^3^) provided by the manufacturer using the same scan parameters: BMD=[raw_attenuation_value×(slope/4096)]+intercept, where slope=411.3990 and intercept=-227.3230.

### Phenotyping using standard X-rays

Armor plate number on the left side, standard length, and ivory vertebrae presence or absence were scored using standard X-rays taken with an UltraFocus X-ray cabinet (Faxitron) using the following parameters: 38 kV and 4.8 s. Standard length was measured using Fiji version 2.0.088 (Schindelin et al. 2012). Ivory vertebrae were scored as present if any vertebrae appeared abnormally bright white and/or deformed.

### QTL mapping

The Bodega Bay, California, USA (BDGB) × Boulton Lake, British Columbia, Canada (BOUL) genetic cross used here—including the F2 fish, genotype information, and linkage map—was previously described by Wucherpfennig et al. 2022. Briefly, a wild-caught BDGB and a wild-caught BOUL stickleback were crossed by *in vitro* fertilization, and F1s were raised in the laboratory. Sperm from a single F1 male was cryopreserved and used to fertilize four F1 females to generate F2s. F2s were raised in the laboratory for 1 year, euthanized, and preserved in 70% ethanol. DNA was extracted from fin clips taken from the F0 founders and F2s and genotyped using an Illumina GoldenGate genotyping array. Intensity data were processed using GenomeStudiov.2011 (Illumina), and uninformative or low-quality SNPs were removed. The linkage map was constructed with tmap version 0.686 (Tennekes 2018) using 343 F2s and 452 markers.

QTL analysis was performed with the scanone function using Haley-Knott regression in R/qtl version 4.2.2 (Broman et al. 2003) with sex as a covariate. A normal model was used for all traits except ivory vertebrae which used a binary model. For each trait, N = 1000 permutation tests were performed to determine the logarithm of the odds (LOD) significance threshold (*α* = 0.05). With the exception of ivory vertebrae which used 335 F2s, all traits were analyzed using 102 F2s. Percent of phenotypic variance explained (PVE) by each significant QTL was calculated using the following equation where N is the number of F2s: <mi>.

### Identifying bone-related genes near peak QTLs

All stickleback genes in the regions surrounding peak QTLs (Supplementary Fig. 2) and their corresponding human orthologs were identified using Ensembl’s BioMart web tool (Smedley et al. 2009). The final set of bone-related genes was then determined by intersecting these human orthologs with the set of human genes annotated with one or more of the following Gene Ontology terms in the AmiGO online database (Ashburner et al. 2000; Carbon et al. 2009; The Gene Ontology Consortium et al. 2023): “ossification,” “bone development,” and “bone remodeling.”

### TNFRSF1B protein alignments and analysis

Human and medaka orthologs for stickleback *Tnfrsf1b* and their corresponding protein sequences and transmembrane helix domains were identified and downloaded using Ensembl’s BioMart web tool (Smedley et al. 2009). BDGB- and BOUL-specific amino acid changes in TNFRSF1B were identified using population-specific SNPs previously published in Roberts Kingman et al. 2021b originally discovered by DNA-sequencing 227 stickleback fish from 132 different populations. Protein alignments were performed using Geneious Prime^®^ 2022.1.1 (https://www.geneious.com) via global alignment with free end gaps with the Blosum62 cost matrix. The SIFT web server (Sim et al. 2012) was used to predict the effect of amino acid changes on protein function.

## Results

### Bone morphometry differences between marine and freshwater stickleback

**Figure 1:**
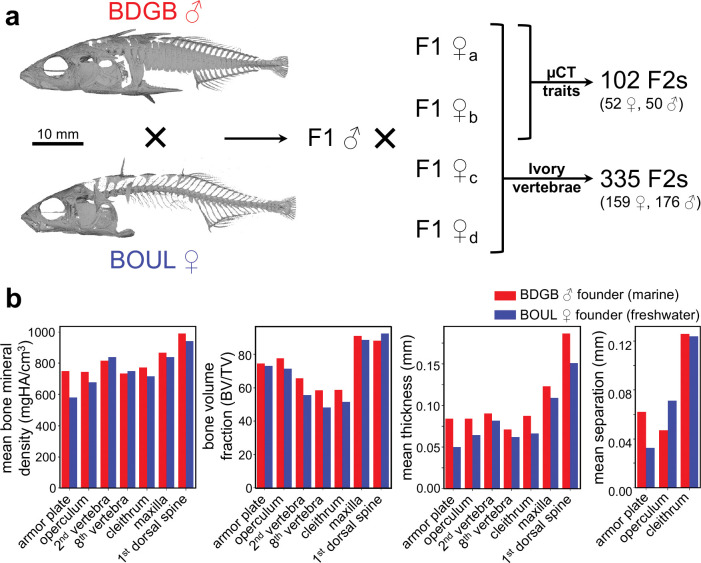
Genetic cross between a marine and freshwater stickleback with divergent bone morphometry traits **a.** Genetic cross between a marine male (BDGB) and freshwater female (BOUL) founder used for QTL mapping. A single F1 male was crossed to four F1 females. 102 F2s from two families were μCT scanned and scored for all traits. These same 102 F2s and 233 additional F2s from two other families were X-rayed and scored for ivory vertebrae. **b.** Quantification of bone morphometry traits for the F0 BDGB and BOUL founders. The two F0 founders were μCT scanned, voxels from each of 7 bones (x-axis) were isolated manually using Dragonfly imaging software, and bone morphometry measurements (y-axis) were calculated using the Dragonfly’s Bone Analysis Extension. For most bones measured, the BOUL freshwater founder has lower mean bone mineral density, bone volume fraction (BV/TV), and mean bone thickness. Note that mean bone separation was calculated only for bones with obvious porous honeycomb-like internal structures.

To study the genetic basis of stickleback internal bone morphometry differences, we used an F2 cross between a wild-caught BDBG marine male stickleback and a wild-caught BOUL freshwater female stickleback (Fig. 1a). This F2 cross was originally generated and genotyped to investigate the genetic basis of dorsal spine number and length variation (Wucherpfennig et al. 2022). We examined the BDGB and BOUL founders of the cross using three-dimensional high-resolution μCT scans and found that the F0 founder fish also differed in several internal bone morphometric traits (Fig. 1b). For the two founder fish, we manually isolated voxels from individual bones of several different types, including: flat porous bones (the armor plate between the first and second pterygiophore and the operculum); individual vertebrae in the spine (the 2nd and 8th); a bone with both a flat porous section and a long dense portion (the cleithrum); and long dense bones (the maxilla and the 1st dorsal spine). The most dramatic differences in bone mineral density, mean bone thickness, and mean bone separation was in the armor plate while the most dramatic difference in bone volume fraction (BV/TV) was in the vertebrae (Fig. 1b).

### Quantitative trait locus mapping of bone traits scorable by μCT

**Figure 2:**
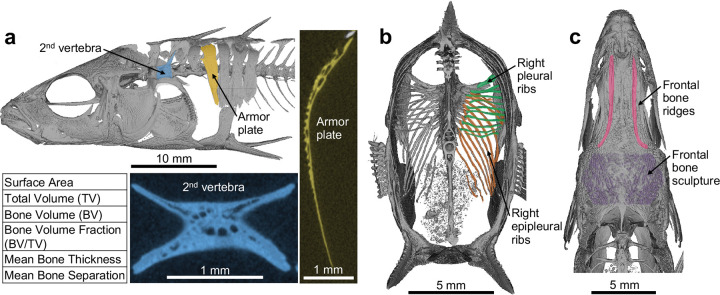
Bone morphometry traits scored via μCT for QTL mapping **a.** (Top left) Lateral view of a representative F2 stickleback fish generated via μCT. The armor plate immediately posterior to the 1st dorsal spine is pseudo-colored in yellow, and the 2nd vertebra is pseudo-colored in blue. (Right) Representative μCT transverse cross section of the armor plate. (Bottom middle) Representative μCT longitudinal cross section of the 2nd vertebra. (Bottom right) Morphometry measurements scored for the armor plate and the 2nd vertebra for QTL mapping. All but bone volume fraction (BV/TV) were standardized by multiple regression against standard length and sex. Mean bone separation was scored for the armor plate only since the 2nd vertebra lacks obvious porous honeycomb-like internal structures. **b.** Posterior view of a transversally-bisected representative F2 stickleback fish generated via μCT. Right pleural rib number (pseudo-colored in green) and right epipleural rib number (pseudo-colored in orange) were counted for QTL mapping. **c.** Dorsal view of the skull of a representative F2 stickleback fish generated via μCT. The prominence of the frontal bone ridges (pseudo-colored in pink) and frontal bone sculpture (pseudo-colored in purple) were scored for QTL mapping on a scale from 1–5, with 5 indicating the greatest prominence.

To examine the possible genetic basis of these internal bone microstructural differences, we also measured bone morphometric traits in armor plates and vertebra of 102 F2 progeny from the cross between BDGB and BOUL (Fig. 1a, Fig. 2a). We focused on the armor plate between the first and second pterygiophore (referred to simply as “the armor plate” for the remainder of this study) and on the 2nd vertebra. We isolated each bone from μCT scans and measured the following: surface area, total volume (TV), bone volume (BV), bone volume fraction (BV/TV), and mean bone thickness (Fig. 2a). Mean bone separation—the mean diameter of the cavities within the bone—was calculated for the armor plate only. With the exception of bone volume fraction (BV/TV), these traits were standardized by multiple regression against standard length and sex.

We also scored several other traits that were easily measurable with μCT scans, including the total number of armor plates, the number of pleural ribs on the right side, the number of epipleural ribs on the right side, the prominence of the ridges on the dorsal side of the frontal bone (referred to as “frontal bone ridges”), and the prominence of texture on the dorsal side of the frontal bone (referred to as “frontal bone sculpture”) (Fig. 2b–c). Numerical traits were quantified by direct counting, and the two frontal bone traits were scored on a semiquantitative scale from 1–5, with 5 indicating the greatest prominence.

**Figure 3:**
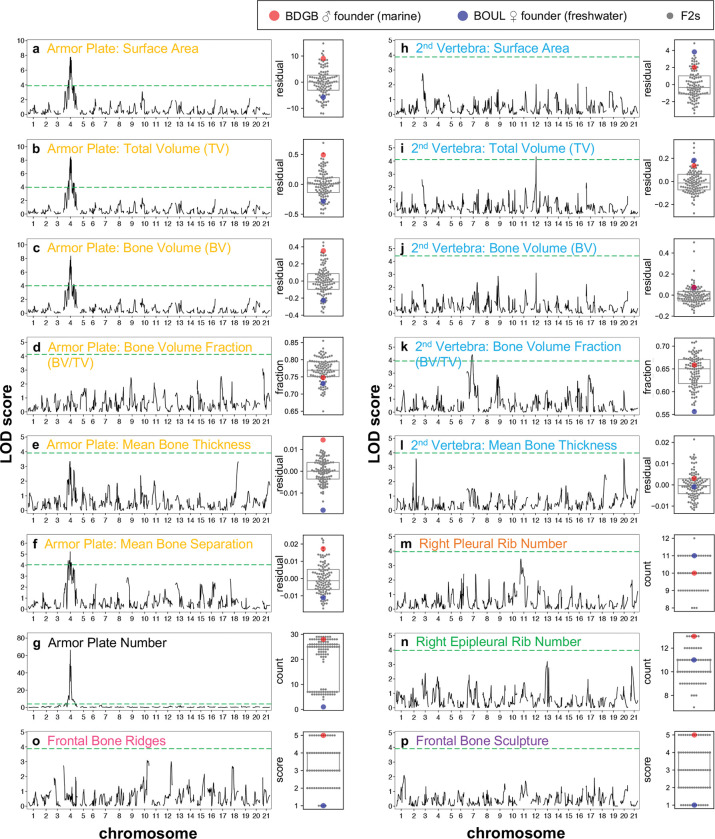
QTL mapping results for bone morphometry and other traits scored by μCT Each panel (**a-p**) shows the QTL scan results (left) and a box plot of the distribution of phenotypes (right) for a single trait, with the title color corresponding to the color scheme in Fig. 2. The QTL scan results for each trait are represented by the logarithm of the odds (LOD) on the y-axis plotted against the chromosomal map position in cM on the x-axis, with the horizontal dashed green line indicating the genome-wide significance threshold (*α* = 0.05) calculated from 1,000 permutation tests. Each box plot represents the distribution of phenotypes observed for that trait, with each gray dot representing the phenotypic value for a single F2 fish and the red and blue dots corresponding to the phenotypic value for the F0 BDGB and BOUL founders, respectively. Armor plate surface area (**a**), total volume (**b**), bone volume (**c**), and mean bone separation (**f**) map to the same region on chromosome 4 as armor plate number (g). 2nd vertebra total volume (**i**) and bone volume fraction (**k**) have QTLs that just pass the genome-wide significance threshold on chromosome 12 and 7, respectively. All other traits do not have significant detected QTLs.

Using a linkage map of 452 markers (Wucherpfennig et al. 2022), we carried out QTL mapping to examine the genetic basis of these bone morphometry, bone number, and frontal bone traits scorable by μCT. We found a strong QTL on chromosome 4 for the following armor plate traits: surface area (LOD: 7.9, PVE: 30.0%), total volume (LOD: 8.6, PVE: 32.3%), bone volume (LOD: 8.5, PVE: 31.8%), and mean bone separation (LOD: 5.2, PVE: 21.0%) (Fig. 3a–c,f, Table 1). Armor plate number has been previously mapped to this same region of chromosome 4 (Colosimo et al. 2004), and this result is replicated for the 102 F2s used in this study (LOD: 66.3, PVE: 95.0%) (Fig. 3g, Table 1). We found only modest QTL peaks for 2nd vertebra traits, with the total volume (LOD: 4.4, PVE: 18.0%) and bone volume fraction (LOD: 4.4, PVE: 18.1%) peaks just passing the significance threshold on chromosomes 12 and 7 respectively (Fig. 3i,k, Table 1). No significant QTL peaks were identified for any other traits, including rib number and frontal bone traits.

### QTL mapping of ivory vertebrae

**Figure 4:**
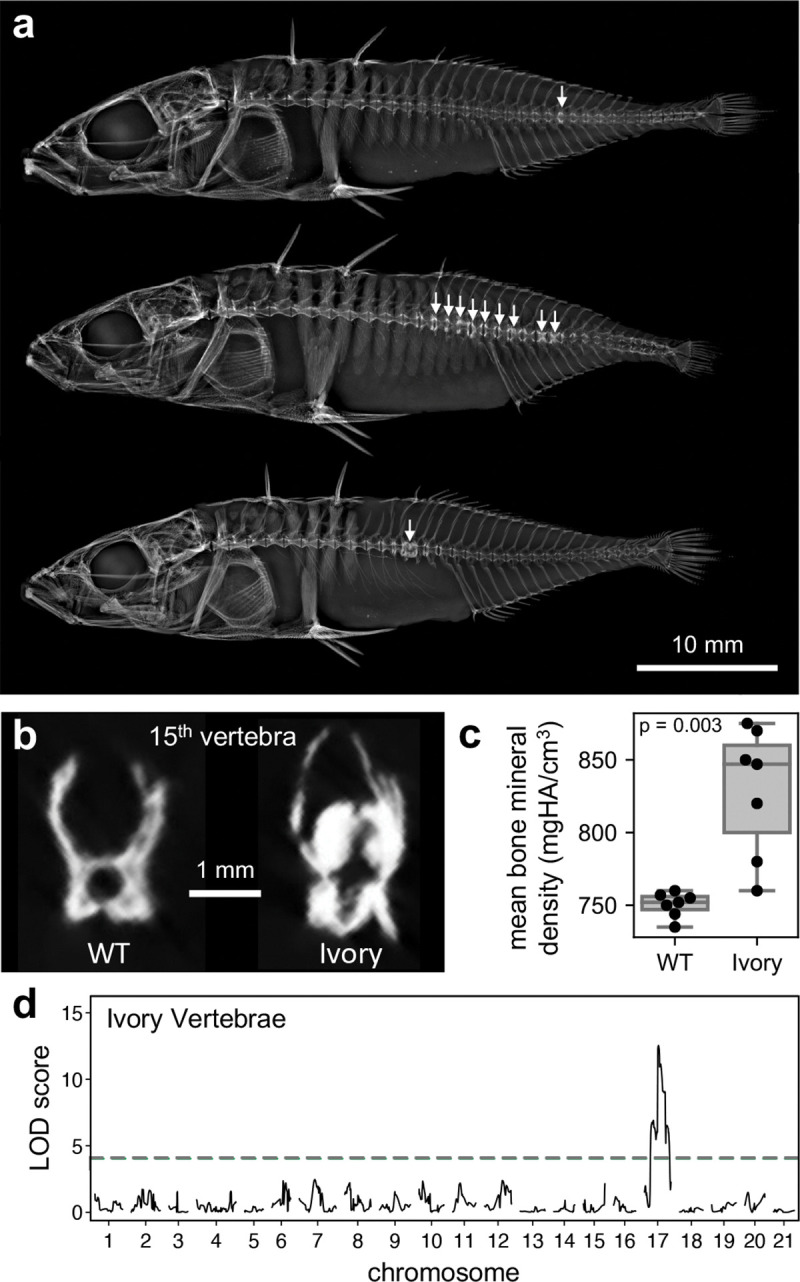
A major-effect QTL for ivory vertebrae on chromosome 17 **a.** Standard X-rays of representative F2 fish with white arrows indicating ivory vertebrae. **b.** Representative μCT transverse cross-sections of a wildtype (WT) and an ivory 15th vertebra. **c.** Quantification of mean bone mineral density (y-axis) for fish with a WT 15th vertebra (N = 7) and fish with an ivory 15th vertebra (N = 7). Ivory vertebrae have statistically significantly higher bone mineral density with a mean of 829 mgHA/cm^3^ compared to 750 mgHA/cm^3^ for WT vertebrae (p = 0.003 by Welch’s t-test). **d.** QTL scan results for ivory vertebrae showing a major-effect QTL on chromosome 17. Logarithm of the odds (LOD) is on the y-axis plotted against the chromosomal map position in cM on the x-axis, with the horizontal dashed line indicating the genome-wide significance threshold (*a* = 0.05) calculated from 1,000 permutation tests.

While analyzing standard two-dimensional X-rays from the same BDGB × BOUL cross, we noted that 28 of 335 F2s have abnormally bright white vertebrae (Fig. 4a). Similar bright white “ivory vertebrae” are also found in X-rays of humans with Paget’s disease, the second most common metabolic bone disease after osteoporosis (Kravets 2018). In the stickleback cross, both F0 founders were unaffected, and the number, location, and brightness of ivory vertebrae was highly variable across F2 progeny (Fig. 4a). μCT transverse cross-sections of the most commonly affected vertebra (vertebra 15, N = 7) showed overgrowth and abnormal morphology of the ivory vertebra compared to WT (Fig. 4b) and a statistically significant increase in mean bone mineral density (mean 829 mgHA/cm^3^ compared to 750 mgHA/cm^3^, p = 0.003 by Welch’s t-test) (Fig. 4c).

We identified a major-effect QTL for ivory vertebra presence/absence on chromosome 17 (LOD: 12.5) that explains 15.8% of the observed phenotypic variance (Fig. 4d, Table 1). The stickleback gene *Tnfrsf1b* is located within this QTL peak and is orthologous to the human gene *TNFRSF11A*, which is associated with susceptibility to Paget’s disease of bone in humans (Hughes et al. 2000; Makaram and Ralston 2021).

## Discussion

Although multiple previous studies have mapped genetic loci controlling the size, shape, and number of skeletal structures in stickleback, this is the first study to our knowledge to map differences in the internal microstructure of individual bones in the stickleback skeleton. Because high resolution μCT imaging and segmentation of individual bones in the skeleton is time- and labor-intensive, we were limited in the total number of animals we could score in the cross. Using phenotypic analysis of 102 F2 animals, we did not detect any significant QTLs for rib number, frontal bone sculpture or ridge prominence, armor plate bone volume fraction or mean bone thickness, or vertebral surface area, bone volume, or mean bone thickness. One potential explanation is that a cross of this size only has sufficient power (>80%) to detect loci explaining 11% or more of the observed phenotypic variance in a trait (Purcell et al. 2003). We hypothesize, however, that many stickleback bone morphometry traits may be influenced by numerous loci each with small phenotypic effect rather than a few loci with large effects, consistent with findings from human GWAS studies of internal bone density (Ralston and Uitterlinden 2010; Richards et al. 2012; Morris et al. 2019; Zhu et al. 2021).

### Armor plate traits

Despite limited animal numbers, our genetic cross did detect significant QTLs for several armor plate and vertebral traits. Previous studies have shown that the number of armor plates in stickleback is controlled in large part by regulatory variation in the *Ectodysplasin* (*Eda*) gene on chromosome 4 (Colosimo et al. 2004; O’Brown et al. 2015). As expected, the absolute number of armor plates also mapped to the *Eda* region in our current cross. Several newly-measured aspects of the internal microstructure of armor plates also showed linkage to the *Eda* chromosome region, including surface area, total volume, bone volume, and mean bone separation (Fig. 3, Supplementary Fig. 1). One simple explanation for these results is that *Eda* plays a key role in plate development, and multiple different aspects of armor plate bone structure change because of pleiotropic consequences due to alterations affecting *Eda* itself. Alternatively, there may be other linked genes near *Eda* that each contribute to different aspects of armor plate structure. A similar phenomenon has previously been reported for defensive spine formation in stickleback, where at least two different genes (*Msx2* and *Stc2a*) that are physically linked within 2 Mb of the *Eda* locus both contribute to genetic variation in length of dorsal and/or pelvic spines (Howes et al. 2017; Roberts Kingman et al. 2021a).

Interestingly, LOD scores along chromosome 4 show somewhat distinctive patterns for the different aspects of armor plate microstructure that we measured in the current study (Supplementary Fig. 1). In particular, while the peak marker for armor plate number and mean bone separation maps within the *Eda* gene itself, the peak marker for armor plate surface area, total volume, and bone volume is ~2.9 Mb downstream in the gene *Integral membrane protein 2A* (*Itm2a*). *Itm2a* is involved in endochondral bone formation (Van Den Plas and Merregaert 2004), and either *Itm2a* or other physically linked genes in the chromosome 4 region may also contribute to one or more of the armor plate microstructure traits we investigated in this study. Indeed, in organisms as diverse as plants, butterflies, and stickleback, linked physical clusters of genes and regulatory elements (“supergenes”) are known to control multiple different aspects of complex flower, wing, and skeletal phenotypes (Schwander et al. 2014; Thompson and Jiggins 2014; Herbert et al. 2024). Our studies add to accumulating evidence that the *Eda*/*Itm2a* region of chromosome 4 is also a supergene region that controls multiple different aspects of armor plate development, defensive spine formation, and tooth and behavioral phenotypes (Erickson et al. 2018; Roberts Kingman et al. 2021a).

### Vertebral porosity

Although the majority of the vertebral bone morphometry traits we measured did not show significant linkage to any chromosome regions, total volume (TV) and bone volume fraction (BV/TV) did show QTLs just above the significance threshold. Bone volume fraction (BV/TV) of the 2nd vertebra—the proportion of the vertebra’s total volume that is mineralized bone rather than cavity—is particularly interesting because of its relationship to osteoporotic changes in humans. Osteoporosis is characterized by increased bone porosity (i.e. lower bone volume fraction), and vertebral fractures are the second most common fracture type in osteoporosis patients (Compston et al. 2019). In humans, women who experience vertebral fractures in their 60s have ~10% lower bone volume fraction (BV/TV) than age-matched women without fractures (Ito et al. 2005). In the stickleback we measured in this study, the mean bone volume fraction (BV/TV) was reduced by 3.3% from 0.664 in F2s with two marine BDGB alleles to 0.631 in F2s with two freshwater BOUL alleles at the peak marker (Table 1). Notably, stickleback genes with human orthologs related to bone development are located near the peak LOD scores for both vertebral traits (Supplementary Fig. 2). These include human genes *CEBP*, *TNFRSF11A*, *ATP6AP1*, *SRC*, and *HSPG2* for total volume, and *BBX*, *ACHE*, *PPP3CA*, *PANX3*, *FREM1*, *MEN1*, *SERPINH1*, *MMP14*, and *RBPJ* for bone volume fraction (BV/TV) of the 2nd vertebra.

While low bone volume fraction (i.e. high porosity) increases risk of bone fracture in humans (Ito et al. 2005), it may be beneficial in freshwater stickleback by helping to achieve neutral buoyancy in freshwater, which has approximately 2.5% lower density than ocean water (Myhre and Klepaker 2009). Further study of stickleback genes within the bone volume fraction QTL may help identify genetic mechanisms that contribute to adaptive variation in skeletal density in different natural environments.

### Genetic basis of ivory vertebrae in stickleback

One unexpected finding in our study was the formation of unusually thickened “ivory vertebrae” in a fraction of F2 fish in the cross. This phenotype was not detected in either of the two F0 founders of the cross and occurred at relatively low incidence in subsequent generations (28 of 335 F2s). Unlike the relatively weak QTL for increased vertebral porosity, the QTL for presence of thickened ivory vertebrae is highly significant, with a LOD score of 12.5. At the peak marker, all but 4 of the 28 fish with ivory vertebrae are homozygous for the freshwater BOUL allele (3 are heterozygous, 1 is homozygous for the marine BDGB allele). Thus, the freshwater BOUL allele acts as a risk allele in the cross, but also shows incomplete penetrance, with only 24 of 79 F2s (30%) homozygous for the freshwater BOUL allele exhibiting ivory vertebrae (Table 1).

The presentation of ivory vertebrate in the stickleback cross is reminiscent of Paget’s disease in humans, which is characterized by excessive bone resorption by osteoclasts that leads to excessive, disorganized bone formation (Makaram and Ralston 2021). The incomplete penetrance of ivory vertebrae in stickleback also parallels Paget’s disease in humans, where an interaction between genetic and environmental factors leads to disease presentation (Numan et al. 2019; Makaram and Ralston 2021; Banaganapalli et al. 2023). Genetic and genome-wide association studies of human Paget’s disease have identified more than a dozen different genes that increase risk of the disease (Makaram and Ralston 2021). One of these genes is *Tumor Necrosis Factor Receptor Superfamily Member 11A* (*TNFRSF11A*), which encodes Receptor Activator of Nuclear Factor Kappa B (RANK) (Hughes et al. 2000). Signaling through RANK is critical for osteoclastogenesis (Kong et al. 1999; Li et al. 2000; Boyle et al. 2003), and the human Paget’s disease mutations appear to be activating rather than inactivating mutations in the gene (Hughes et al. 2000; Alonso et al. 2021).

Interestingly, a stickleback homolog of this gene (*Tumor necrosis factor receptor superfamily member 1B*, *Tnfrsf1b*) is located within the highly significant chromosome 17 QTL for ivory vertebrae, less than 1 Mb away from the peak marker. The freshwater BOUL version of this gene shows 20 SNPs compared to the marine BDGB version based on sequence surveys of wild stickleback (Roberts Kingman et al. 2021b). Four of the BOUL SNPs are nonsynonymous variants that are predicted to alter two amino acids at positions otherwise conserved between marine stickleback and the distantly related medaka fish outgroup (Supplementary Fig. 3). As in human patients with Paget’s disease variants of *TNFRSF11A*, neither of these changes in *Tnfrsf1b* are predicted to be inactivating mutations (Ng and Henikoff 2003; Sim et al. 2012), but they could lead to more subtle changes in protein function. Alternatively, regulatory changes at the *Tnfrsf11b* locus, or coding or regulatory changes in other closely linked genes on chromosome 17, may contribute to the development of ivory vertebrae in stickleback.

Stickleback are often studied because of their usefulness for evolutionary and ecological questions. We show here that stickleback may also serve as valuable models for genetic studies of skeletal traits and disease that vary across a broad range of other animals, including humans.

## Supplementary Material

Supplement 1

Supplement 2

Supplement 3

Supplement 4

## Figures and Tables

**Table 1:** Significant QTLs

					Phenotype mean ± standard error		
	Chr	Position	LOD	PVE	MM	MF	FF

**Armor plate traits**							
Surface area	4	15,712,088	7.9	30.0	4.12 ± 0.90	1.09 ± 0.67*	−4.08 ± 0.68**••
Total volume (TV)	4	15,712,088	8.6	32.3	0.198 ± 0.044	0.043 ± 0.027*	−0.179 ± 0.027**••
Bone volume (BV)	4	15,712,088	8.5	31.8	0.143 ± 0.033	0.035 ± 0.021*	−0.135 ± 0.021**••
Mean bone separation	4	12,795,421	5.2	21.0	−0.0058 ± 0.0014	0.0012 ± 0.0011**	0.0060 ± 0.0020**•
Armor plate number	4	12,795,421	66.3	95.0	26.63 ± 0.49	25.18 ± 0.33*	6.57 ± 0.16**••

**2nd vertebra traits**							
Total volume (TV)	12	5,248,996	4.4	18.0	0.058 ± 0.035	−0.005 ± 0.011	−0.034 ± 0.015*
Bone volume fraction (BV/TV)	7	5,000,365	4.4	18.1	0.664 ± 0.005	0.641 ± 0.005*	0.631 ± 0.007**

**Other traits**					**N with phenotype / total**		
Ivory vertebrae	17	6,118,201	12.5	15.8	1/80	3/176	24/79**••

All significant QTLs that exceed the genome-wide significance threshold are shown, along with their respective chromosome (Chr) and physical position (Position) in the GAculeatus_UGA_version5 genome assembly, maximum logarithm of the odds (LOD) score, and corresponding percent of phenotypic variance explained (PVE). For most traits, the phenotype mean and standard error for fish in each genotype class is reported (MM homozygous for marine BDGB allele; MF heterozygous; FF homozygous for the freshwater BOUL allele).

Note that for the 2nd vertebra traits total volume (TV) and bone volume fraction (BV/TV), phenotype means were computed using the nearest marker to the peak since the peak LOD score is imputed. Also note that since ivory vertebrae is a binary trait (either present or absent), phenotype means are not reported. Instead, the number of animals with ivory vertebrae and the total number of fish in that genotype category are shown.

Statistical tests were performed using Welch’s t-test.

* Significantly different from MM mean (*p* < 0.05)

** Highly significantly different from MM mean (*p* < 0.0005)

• Significantly different from MF mean (*p* < 0.05)

•• Highly significantly different from MF mean (*p* < 0.0005)

## Data Availability

All linkage map, genotype, and phenotype data used for QTL mapping are provided as Supplementary Files accompanying this manuscript. Supplementary File 1 contains the linkage map and genotype data for all F2 stickleback analyzed in this study. The first row lists linkage map marker IDs, named according to their physical position in the gasAcu1 genome assembly along with a unique identifier (e.g., the marker “chrI.1549902.SNP0006” corresponds to chromosome 1 at position 1,549,902 in gasAcu1, with SNP0006 as the unique marker ID). The second row provides the genetic position of each marker in centimorgans. All subsequent rows contain genotype information for individual F2 fish (IDs formatted as “DK184.013”). Genotypes are coded as “A” (homozygous for the freshwater BOUL allele), “B” (homozygous for the marine BDGB allele), and “H” (heterozygous, carrying one BOUL and one BDGB allele at that marker). Supplementary File 2 contains ivory vertebrae status (0 indicates absence, 1 indicates presence), bone morphometric measurements, armor plate number, rib counts, and frontal bone phenotypes for all F2 stickleback. Both the original raw measurements (units: mm, mm^2^, and mm^3^) and the normalized residuals used for QTL mapping are provided for bone morphometric phenotypes. Residuals were calculated after multiple regression against standard length and sex. Raw and normalized values are included as separate columns (e.g. “armor-plate_total-volume” and “armorplate_total-volume.res” correspond to raw and residual values, respectively). Supplementary File 3 provides a look-up table for converting select coordinates between the gasAcu1 and GAculeatus_UGA_version5 genome assemblies. Only coordinates used in Table 1 and Supplementary Figures 1 and 2 are included. This file is provided because the linkage map marker names originally described in Wucherpfennig et al. 2022 and reproduced here in Supplementary File 1 were generated using gasAcu1 coordinates, whereas the Table and Figures have been updated to reflect the improved GAculeatus_UGA_version5 assembly. Supplementary material will be available at *G3* online after publication.
