# Supplementary material for: Genetic analysis of bone morphometry and ivory vertebrae in threespine stickleback
Source: bioRxiv. 2026 Jun 5:2026.04.13.718284. Originally published 2026 Apr 14. Preprint. [Version 2] doi: 10.64898/2026.04.13.718284 (PMC13104931; doi:10.64898/2026.04.13.718284)
Supplement: Supplement 4 [file NIHPP2026.04.13.718284v2-supplement-4.pdf]

bioRxiv preprint doi: https://doi.org/10.64898/2026.04.13.718284; this version posted June 5, 2026. The copyright holder for this preprint (which was not certified by peer review) is the author/funder, who has granted bioRxiv a license to display the preprint in perpetuity. It is made available under a CC-BY 4.0 International license.

Supplementary Figures

For Behrens *et al.* 2026, Genetic analysis of bone morphometry and ivory vertebrae in threespine stickleback

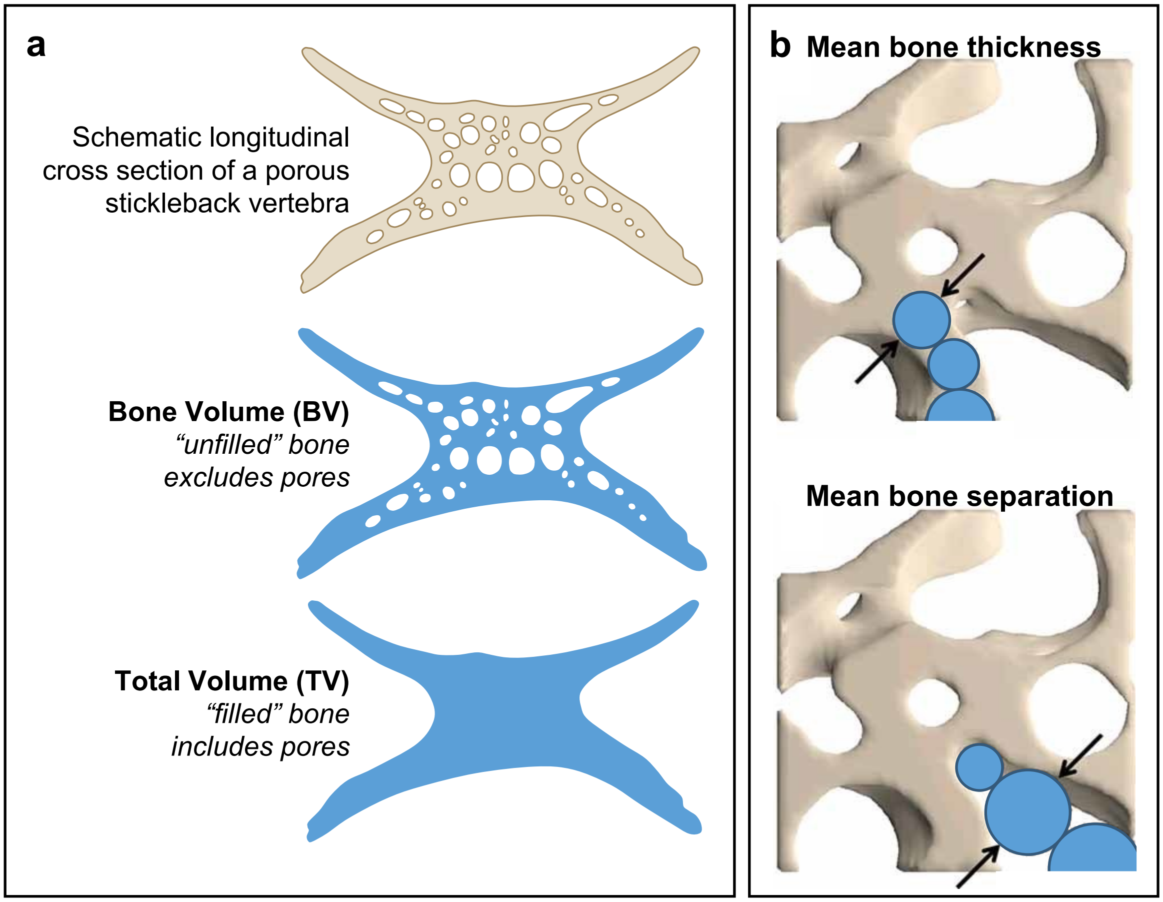

Supplementary Figure 1: Schematic representation of µCT-derived bone morphometry measurements

a. Schematic 2D representation of the algorithm used to compute the bone volume (BV) of the unfilled bone-of-interest and the total volume (TV) of the filled bone-of-interest. Bone volume fraction (BV/TV) (not pictured) is the ratio of the unfilled bone volume to the filled total volume. Surface area (not pictured) is the external surface area of the unfilled bone.

b. Mean bone thickness and mean bone separation are quantified by fitting spheres within the mineralized structure (for mean bone thickness) or within the porous space (for mean bone separation). The mean diameter of the fitted spheres is reported as mean bone thickness or separation. (Adapted from Fig. 6 of Bouxsein *et al.* 2010, originally courtesy of Andres Laib, Ph.D., Scanco Medical AG)

bioRxiv preprint doi: https://doi.org/10.64898/2026.04.13.718284; this version posted June 5, 2026. The copyright holder for this preprint (which was not certified by peer review) is the author/funder, who has granted bioRxiv a license to display the preprint in perpetuity. It is made available under a CC-BY 4.0 International license.

Supplementary Figures

For Behrens *et al.* 2026, Genetic analysis of bone morphometry and ivory vertebrae in threespine stickleback

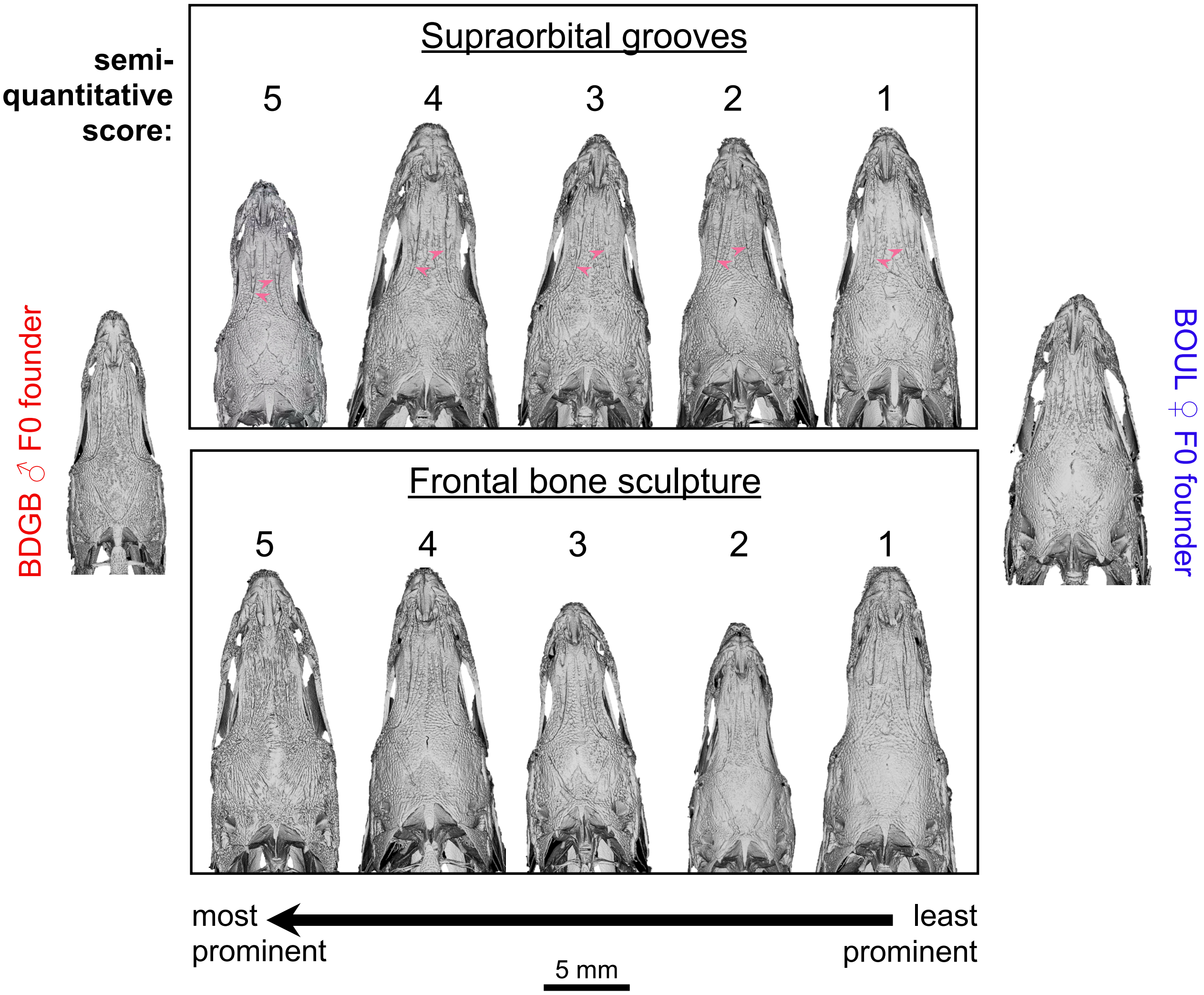

Supplementary Figure 2: Semi-quantitative phenotype categories for supraorbital grooves and frontal bone sculpture

Dorsal view of representative F2 stickleback fish generated via µCT demonstrating the semi-quantitative scoring system for frontal bone traits. Supraorbital grooves (**top, arrowheads**) and frontal bone sculpture (**bottom**) were scored for QTL mapping on a scale from 1-5, with 5 indicating the greatest prominence.

IDs for the F2s shown above are as follows, listed from left to right:

Supraorbital grooves: DK194.007, DK185.125, DK185.003, DK185.131, DK185.005

Frontal bone sculpture: DK185.119, DK185.160, DK185.159, DK194.025, DK185.012

bioRxiv preprint doi: https://doi.org/10.64898/2026.04.13.718284; this version posted June 5, 2026. The copyright holder for this preprint (which was not certified by peer review) is the author/funder, who has granted bioRxiv a license to display the preprint in perpetuity. It is made available under a CC-BY 4.0 International license.

Supplementary Figures

For Behrens *et al.* 2026, Genetic analysis of bone morphometry and ivory vertebrae in threespine stickleback

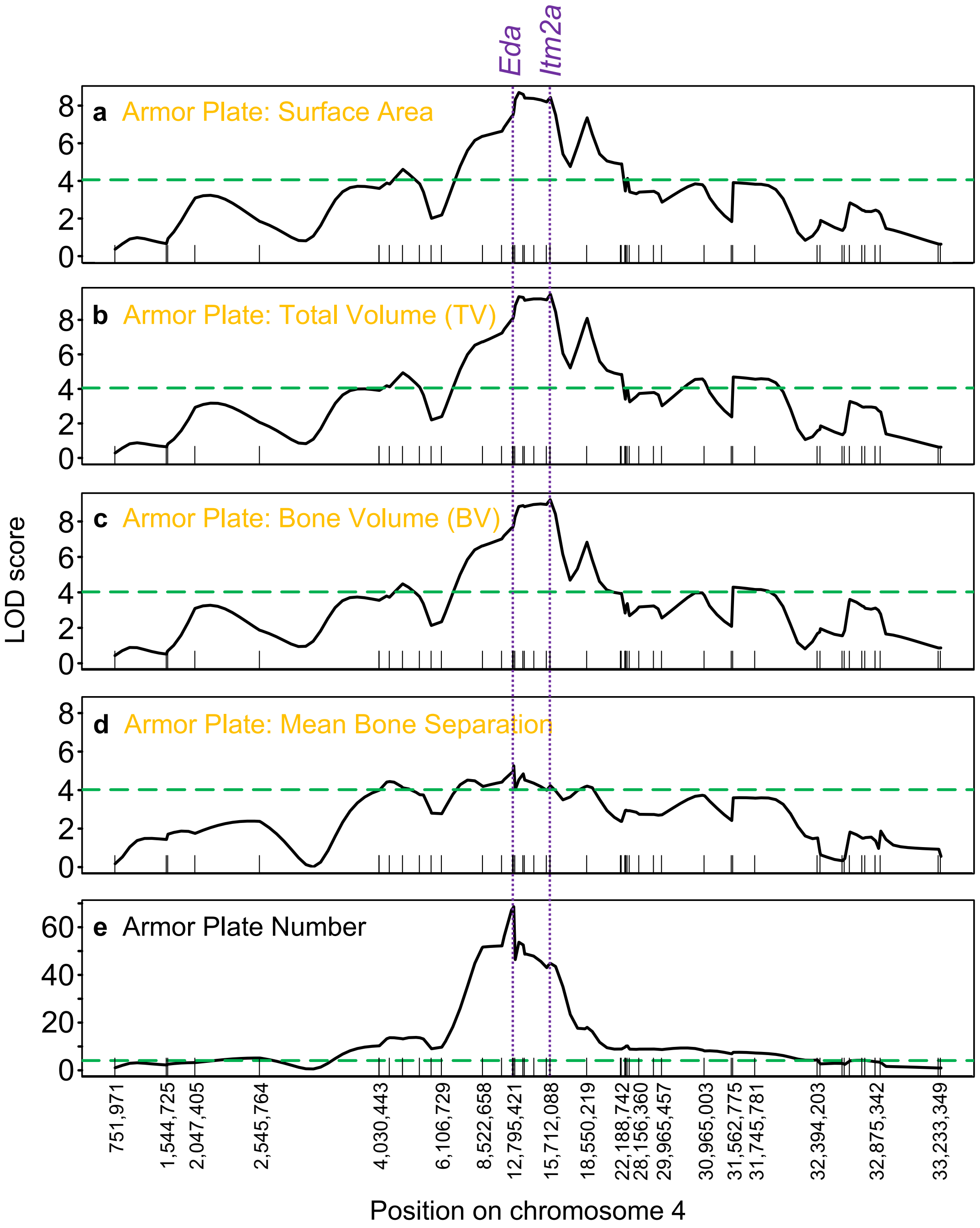

Supplementary Figure 3: Candidate region genomic scans for armor plate QTLs

Single-chromosome QTL scan results for armor plate bone morphometry traits on chromosome 4 with significant QTLs. Dotted purple vertical lines spanning all plots indicate the position of the genes *Eda* and *Itm2a*. Note that the peak marker for total volume (**b**) and bone volume (**c**) is within *Itm2a*, but the peak marker for mean bone separation (**d**) and plate number (**e**) is near *Eda*. The peak LOD score for surface area (**a**) falls between *Eda* and *Itm2a*. Chromosome 4 coordinates (in the GAculeatus_UGA_version5 genome assembly) for select markers are shown on the y-axis, and plots were generated as described in the Fig. 3 legend.

bioRxiv preprint doi: https://doi.org/10.64898/2026.04.13.718284; this version posted June 5, 2026. The copyright holder for this preprint (which was not certified by peer review) is the author/funder, who has granted bioRxiv a license to display the preprint in perpetuity. It is made available under a CC-BY 4.0 International license.

Supplementary Figures

For Behrens *et al.* 2026, Genetic analysis of bone morphometry and ivory vertebrae in threespine stickleback

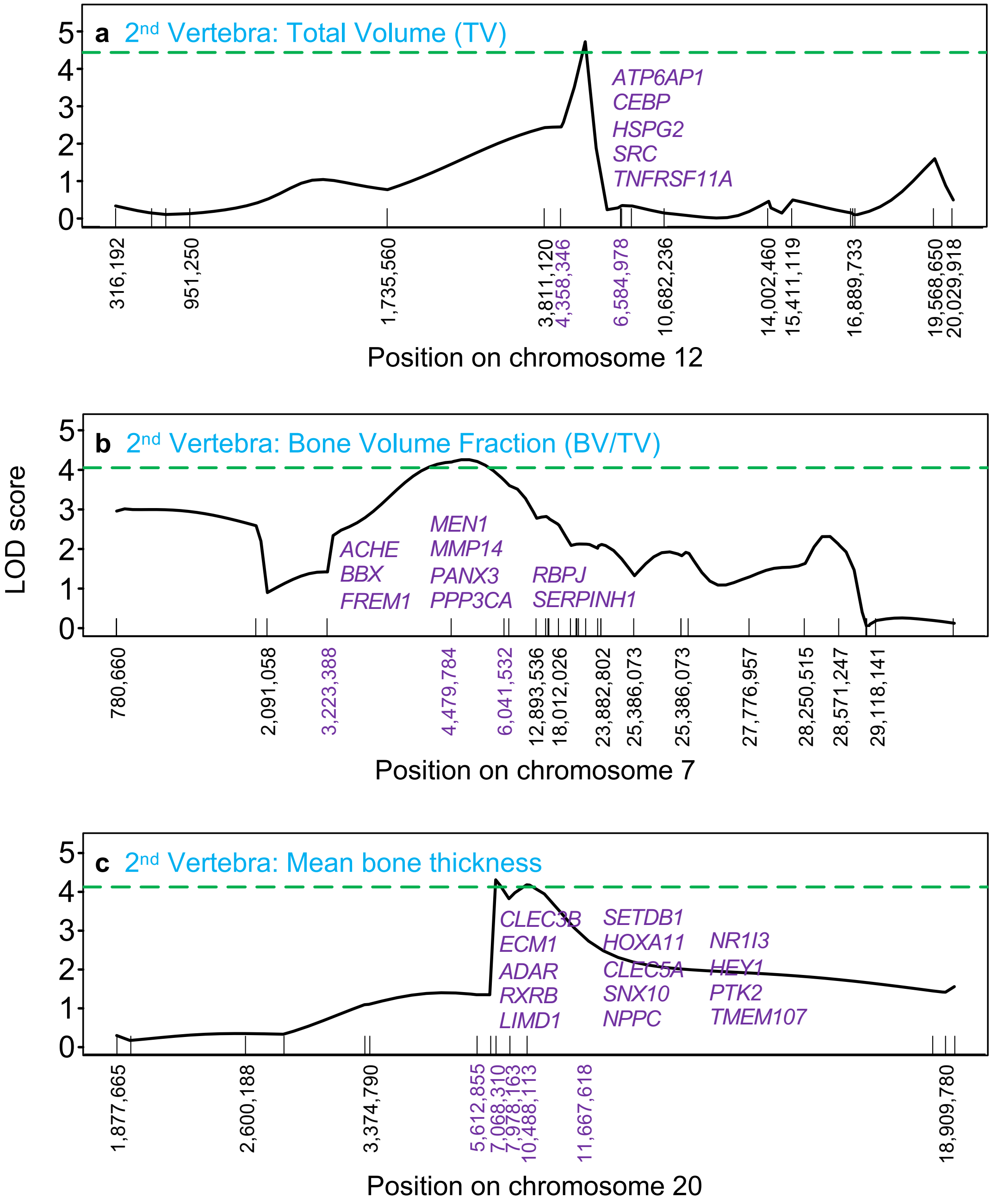

Supplementary Figure 4: Candidate skeletal genes in 2nd vertebra QTL regions

Single-chromosome QTL scan results for 2nd vertebra traits with significant QTLs. Chromosome 12 (**a**), 7 (**b**), or 20 (**c**) coordinates (in the GAculeatus_UGA_version5 genome assembly) for select markers are listed on the y-axis. The coordinates in purple represent the candidate region used to identify candidate genes. Bone-related human orthologs for stickleback genes within the candidate region are shown in purple text on each plot. Plots were generated as described in the Fig. 3 legend.

bioRxiv preprint doi: https://doi.org/10.64898/2026.04.13.718284; this version posted June 5, 2026. The copyright holder for this preprint (which was not certified by peer review) is the author/funder, who has granted bioRxiv a license to display the preprint in perpetuity. It is made available under a CC-BY 4.0 International license.

Supplementary Figures

For Behrens *et al.* 2026, Genetic analysis of bone morphometry and ivory vertebrae in threespine stickleback

```
BDGB    ---------- ---------- ---------- --MKEILVVL LLLHVQTLKV CSQPYQADSD GNCLNSTTEY 38
BOUL    ---------- ---------- ---------- --........ .......... .......... .......... 38
Medaka  MRKYSLRFHT PPDLPVIFIH TTPVNSVFTK EA.GDLF... ...S...T.- ------.N.H ESIC.EN... 63

BDGB    LLDDTNLCCK KCPPGHRQSE KCSETTETVC ERCQPRLFIE SWNYSPNCFS CTQCKEKKGL KEAQVCSPTE 108
BOUL    .......... .......... .......... .......... I........P .......... .......... 108
Medaka  .K.G.D.... ..Q..YHLG. H...NK.... .P.KSNTYL. N...AQ.... .KI.NPR.L. RYE.N.TL.K 133

BDGB    NSRCVCQPGM FCSMGFDDPF CTACSKYRSC RPGFGVSMQG TADRDVRCTR CPSGTFSDTL SSTTRCLSHA 178
BOUL    .......... .......... .......... .......IP. .......... .......... .......... 178
Medaka  .AV...E.ET ..AI-LLK.E .S..KR..K. P..Q...V.. .PSS..K.QK ..N....SIS .NSEK.KP.T 202

BDGB    DCNGRVVAKK GNATSDNVCE PRAFAHTARP PTLTRRPMDA LGFVATRTVS ATSDSEAARG QTEASLSIST 248
BOUL    .......... .......... .......... .......... .......... .......... .......... 248
Medaka  ..K..ALV.. .D.I...I.. DE.-PKPLKR A.PRAPVVIV .TSTEANNPG T.I.FTTT.. VKGFTQTSN. 271

                                       transmembrane helix domain
BDGB    SYSRPEISPR PPDPPPDQNT ATGKVLAAVI AGVIGLLLFC ISIVLVFFCK PVRKKDDAAR FHPKVDGNGS 318
BOUL    .......... .......... .......... .......... .......... .......... .......... 318
Medaka  FV.FESS.ST KSPHTTK.PD IKPV.I.SSV V.IFF...TF V.--.L..Y. --.RRT.S.K L.....A..N 337

BDGB    CHRVDNLDQD YLGETYLNYI KVPTPEHQCL LQRGETCSDH SQCSNNTDTL TQTDGSSSQE SVGPLQSTVA 388
BOUL    .......... .......... .......... .......... .......... .......... .......... 388
Medaka  .ENGGKIVQR HEV.RQKMGL ---.S.Q... .GKS.AG.EE .....SS..S .KP.NFI.N. P-ST.L.KSD 403

BDGB    LHSPLSVPSD PMTLLSHTEA ATPQPNVPTQ STSQPTSPQV ISPVTANPHV NVNITFHIEN RSCGTPSVTP 458
BOUL    .......... .......... .......... .......... .......... .......... .......... 458
Medaka  FNN.IFAL.E ......NP.. V....SI.A. PS.......I .....DR... .....V..G. G.YQ.--.N. 471

BDGB    TDLMQVDPKL PFGEEEESFS IPHQEAGKQS LMSVQESDSY RA*------- -------            501
BOUL    .......... .......... .......... .......... ...------- -------            501
Medaka  I.TR.AECQ. ..E..DW.V. T.K..E.E.T CE..P..GAN STYYIPKQFA TCKQAE*            528
```

Supplementary Figure 5: TNFRSF1B amino acid differences between BDGB and BOUL stickleback

Protein alignment of TNFRSF1B for the BDGB marine stickleback, BOUL freshwater stickleback, and outgroup Japanese medaka. Amino acid differences between BDGB and BOUL stickleback are underlined and highlighted in yellow. Two of the four differences are conserved to medaka. The transmembrane helix domain is indicated by a gray bar.

The BDGB and BOUL stickleback protein sequences are based on ENSGACP00000029144.1 (transcript ID ENSGACT00000068214.1) and modified to incorporate amino acid changes resulting from population-specific SNPs identified from previously published DNA-sequencing data in Roberts Kingman *et al.* 2021b. The Japanese medaka protein sequence is ENSORLP00000005099.2 (transcript ID ENSORLT00000005100.2).
